# LCT-3d Induces Oxidative Stress-Mediated Apoptosis by Upregulating Death Receptor 5 in Gastric Cancer Cells

**DOI:** 10.3389/fonc.2021.658608

**Published:** 2021-04-16

**Authors:** Menglin Wang, Xinxin Wu, Lu Yu, Zi-yun Hu, Xiaobo Li, Xia Meng, Chun-tao Lv, Gi-Young Kim, Yung Hyun Choi, Zhengya Wang, Hai-Wei Xu, Cheng-Yun Jin

**Affiliations:** ^1^ Key Laboratory of Advanced Technology for Drug Preparation, Ministry of Education, School of Pharmaceutical Sciences, Zhengzhou University, Zhengzhou, China; ^2^ Department of Marine Life Sciences, Jeju National University, Jeju, South Korea; ^3^ Department of Biochemistry, College of Oriental Medicine, Dong-Eui University, Busan, South Korea; ^4^ State Key Laboratory of Esophageal Cancer Prevention & Treatment, Zhengzhou University, Zhengzhou, China

**Keywords:** LCT-3d, DR5, reactive oxygen species, Nrf2, apoptosis, gastric cancer

## Abstract

Gastric cancer is a global health problem. In this study, we investigate the role of a novel Indole derivative, named LCT-3d, in inhibiting the growth of gastric cancer cells by MTT assay. The Western blotting results showed that LCT-3d modulated the mitochondrial-related proteins and Cleaved-Caspases 3/9, to induce cell apoptosis. The up-regulation of Death receptor 5 (DR5) in MGC803 cells was observed with LCT-3d treatment. Knockdown of DR5 on MGC803 cells partially reversed the LCT-3d-induced mitochondrial apoptosis. The level of Reactive Oxygen Species (ROS) in MGC803 cells was increased with LCT-3d treatment and could be blocked with the pretreatment of the ROS inhibitor N-Acetylcysteine (NAC). The results demonstrate that the elevating ROS can up-regulate the expression of DR5, resulting in apoptosis *via* mitochondrial pathway. Although the nuclear factor erythroid-2 related factor 2 (Nrf2) pathway served an important role in protecting gastric cancer cells against the injury of ROS, it can’t reverse LCT-3d-induced cell apoptosis. Taken together, our study showed that LCT-3d induced apoptosis *via* DR5-mediated mitochondrial apoptotic pathway in gastric cancer cells. LCT-3d could be a novel lead compound for development of anti-cancer activity in gastric cancer.

## Introduction

Gastric cancer remains the third leading cause of death in spite of the decreasing incidence and mortality (1). Many people in China are diagnosed with gastric cancer every year, and once diagnosed, most of them are in the advanced stage with metastasis (2). Currently, chemotherapy for gastric cancer is limited due to serious side effects and low efficacy (3). The development of drug resistance in tumors is a major obstacle of chemotherapy (4–7). Hence, developing novel effective strategies for treatment of gastric cancers is warranted.

Drugs with Indole skeleton shows good efficacy for cancer therapy (8), such as vincristine (9), Indole-3-carbinol (I3C) (10), BPR0L075 (11), as well as JKA97 (12). All of them have exhibited good effects on anti-tumor through inducing cell apoptosis *via* a variety of signaling pathways.

**Figure 1:**
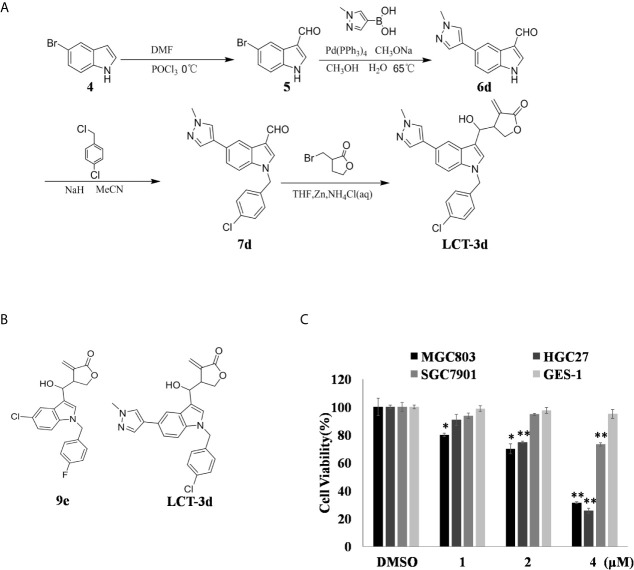
LCT-3d inhibited the cell proliferation of gastric cancer cells. **(A)** LCT-3d synthetic procedure. **(B)** Structure of LCT-3d. **(C)** Cytotoxic effect of LCT-3d on gastric cancer cells measured by MTT assay. Cells were treated with increasing concentration of LCT-3d for 48 h. *P < 0.05, **P < 0.01, significantly different compared with control.

In the previous work, we synthesized a potent Lysine (K)-Specific Demethylase 1A (LSD1) inhibitor, named LCT-9e (**Figure 1B**), which effectively inhibits the macrophages (THP-1 cells) growth (13). LCT-9e is the first irreversible LSD1 inhibitor, which is not derived from monoamine oxidase inhibitors. Hence, LCT-9e was chosen to be a lead compound for optimization. Unexpectedly, when the heterocyclic group was introduced to C-5 position of the Indole, the LSD1 inhibitors such as compound LCT-3d kept the anti-proliferation activity in gastric cancer cells with low cytotoxicity. In this study, we explored the role of LCT-3d in gastric cancer cells and its molecular mechanism. The results suggested that LCT-3d induced the generation of Reactive Oxygen Species (ROS), which subsequently led to gastric cancer cell apoptosis *via* up-regulating the expression of Death receptor 5 (DR5). Meanwhile, LCT-3d activated the nuclear factor erythroid-2 related factor 2 (Nrf2) pathway, which attenuated LCT-3d-induced cell apoptosis. This is the first report on Indole derivative in inducing apoptosis in gastric cancer cells through DR5-mediated pathway.

## Materials and Methods

### Reagents and Antibodies

LCT-3d was synthesized in our group and was dissolved in DMSO. RPMI-1640, Fetal bovine serum (FBS), and penicillin-streptomycin were purchased from HyClone (Victoria, Australia). DCFH-DA (2, 7-dichlorodihydrofuorescein diacetate), the Nuclear and Cytoplasmic Protein Extraction Kit, and Annexin V- FITC Apoptosis Detection Kit were purchased from Beyotime Biotechnology (Shanghai, China). The primary antibodies for Caspase 3 (sc-7272), PARP (sc-7150), DR5 (sc-65314), Caspase 9 (sc-7885), Bim (sc-11425), Bad (sc-8044), Bax (sc-493), DR4 (sc-7863), and β-actin (sc-1615) were purchased from Santa Cruz Biotechnology (Santa Cruz, CA, USA). Bid (#2002), Bcl-xL (#2764), and XIAP (#14334) were purchased from Cell Signaling Technology (Danvers, MA, USA). The secondary antibodies anti-goat, anti-rabbit, and anti-mouse were purchased from Bioss (Shanghai, China). The pan-Caspase inhibitor Z-VAD-FMK was purchased from Selleck. The Nrf2 inhibitor ML385 was purchased from MCE (NJ, USA). The ECL (enhanced chemiluminescence) kit was purchased from Thermo Fisher (Waltham, MA, USA). Anti-DR5 (ab1675) antibody for flow cytometry was purchased from Abcam (Cambridge, MA, USA). NAC (N-acetyl-L-cysteine), MTT [3-(4, 5-dimethylthiazol-2-yl) - 2, 5-diphenyltetra- zolium bromide], and JC-1 fluorescent dye were purchased from Sigma-Aldrich (St. Louis, MO, USA).

### Cell Lines and Culture

Gastric cancer cells MGC803, HGC27, SGC7901 and normal gastric cells GES-1 were from the American Type Culture Collection. These cells were cultured in cell medium supplemented with 10% FBS in an incubator with 5% CO_2_ at 37°C. The DR5 knockout cell line of MGC803 was previously constructed by our team (14).

### Cell Viability Assay

The viability of gastric cancer cells and GES-1 cells were examined by MTT assay. After cells were incubated with LCT-3d for 48 h, 20 μl MTT (5 mg/ml) was added into each well. Subsequently, cells were incubated for another 2–4 h, the cell culture medium was removed, and then 150 μl DMSO was added into each well. Optical density for MTT assay was detected at 490 nm with a microplate reader (Synergy H1, BioTek, VT, USA). The dose of DMSO has no effect on cell viability in all groups of cells in this study.

### Apoptosis Analysis

MGC803 and HGC27 cells were treated with LCT-3d at different concentrations. After incubating for 48 h, the cells were collected and stained with Annexin V and PI for 20 min. Next, cells were measured by a flow cytometer (BD LSRFortessa™ Cell Analyzer, Becton, Dickinson and Company, NJ, USA). The data was quantified by the FlowJo software.

### Measurement of Mitochondrial Membrane Potential (MMP, ΔΨ)

MGC803 and HGC27 cells were treated with LCT-3d at different concentrations. After incubating for 48 h, the cells were collected and then incubated with JC-1 (2.5 μg/ml) for 10 min at 37°C. Next, cells were measured by a flow cytometer. The data was quantified by the FlowJo software (15).

### Analysis of DR5 Expression by a Flow Cytometer

Wild type MGC803 and DR5^−/−^ MGC803 cells were seeded in a six-well plate and treated with LCT-3d for 24 h. The cells were collected and fixed by 1% paraformaldehyde at 4°C for 30 min. Then the cells were incubated with normal goat serum, DR5 antibody, and Goat anti-mouse IgG successively. Next, the cells were measured by a flow cytometer. The data was quantified by FlowJo software.

### Measurement of ROS

MGC803 and HGC27 cells were treated with LCT-3d for indicated time. Then cells were collected and stained with DCFH-DA for 20 min. Next, the cells were measured by a flow cytometer. The data was quantified by FlowJo software.

### Total/Nuclear Protein Extraction

MGC803 cells were treated with LCT-3d for indicated time. Then the cells were collected and proteins were extracted according to instruction of the Nuclear and Cytoplasmic Protein Extraction Kit. The protein concentration was determined by the micro-BCA protein assay kit.

### Western Blotting

MGC803 and HGC27 cells were treated with agents for indicated time. The total proteins were collected and quantified by the micro-BCA protein assay kit (P0012, Beyotime, Shanghai, China). Then the proteins were subjected to immunoblotting as previously described (16).

### Immunofluorescence

Cells were treated with agents for indicated time, then immunofluorescence analysis was performed as reported previously (17).

### Statistical Analysis

All experiments were repeated at least three times. The data were presented as mean ± SD. Differences between the experimental groups were determined by paired or unpaired Student’s t test, * and ** represent *P* < 0.05 and *P* < 0.01, respectively.

## Results

### LCT-3d Reduced the Cell Viability of Human Gastric Cancer Cells but Not Normal Cells

The process of synthesize LCT-3d as shown in **Figure 1A**. The effect of LCT-3d on three human gastric cancer cells (MGC803, HGC27, SGC7901) and normal cell GES-1 were evaluated by MTT assay. The results showed that the viability of MGC803 and HGC27 cells were reduced in a dose-dependent manner after treatment with LCT-3d (**Figure 1C**). In addition, MGC803 and HGC27 cells exhibited more sensitivity to LCT-3d than SGC7901 cells, with the inhibitory rate of 68.83 and 74.41% at 4 μM, respectively. LCT-3d showed little inhibition on the viability of GES-1 cells after treatment with LCT-3d. These results demonstrated that LCT-3d decreased the growth of human gastric cancer cells but not normal cells.

### LCT-3d Induced Cell Apoptosis Dependent on Caspases

**Figure 2:**
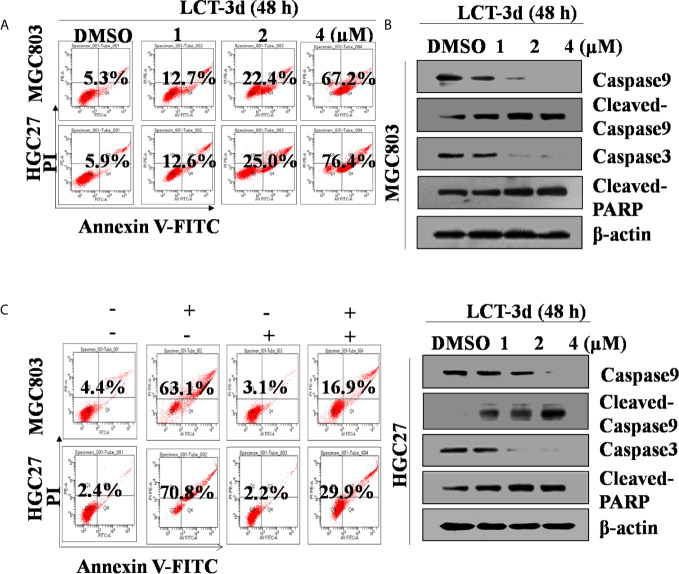
LCT-3d triggered Caspase mediated apoptotic pathway in gastric cancer cells. **(A)** MGC803 cells and HGC27 were cells treated with various concentrations of LCT-3d for 48 h and apoptosis analyzed by flow cytometry. **(B)** Cells were treated as in **(A)** and the expression of Cleaved-Caspase and Cleaved PARP was analyzed by Western blotting. **(C)** MGC803 cells and HGC27 cells were pretreated with a pan-Caspase inhibitor, Z-VAD-FMK (100 μM) for 1 h, followed by incubation with LCT-3d (4 μM) for 48 h. Flow cytometric analysis on the effect of Z-VAD-FMK on LCT-3d-induced cells apoptosis.

In order to determine whether LCT-3d induced cell apoptosis, the apoptotic rate of MGC803 and HGC27 cells was detected by flow cytometry. The apoptotic rate was increased in a concentration-dependent manner in both cells (**Figure 2A**). Then some Caspase-related proteins were tested (**Figure 2B**). Western blotting results demonstrated that LCT-3d promoted the expression of Cleaved-PARP and Cleaved-Caspase 3/9 in MGC803 and HGC27 cells.

To further confirm whether Caspase family proteins regulated LCT-3d-induced apoptosis, the cells were pretreated with a pan-Caspase inhibitor Z-VAD-FMK (100 μM) for 1 h, and subsequently incubated with LCT-3d (4 μM) for an additional 48 h. The flow cytometry analysis showed that pretreatment with Z-VAD-FMK (100 μM) partially reversed LCT-3d-induced apoptosis in MGC803 and HGC27 cells (**Figure 2C**). These results suggested that LCT-3d induced apoptosis of the gastric cancer cells through a Caspase-dependent manner.

### LCT-3d Regulated the Expression of Bcl-2 Family Proteins and Reduced the Mitochondrial Membrane Potential (MMP/ΔΨ)

**Figure 3:**
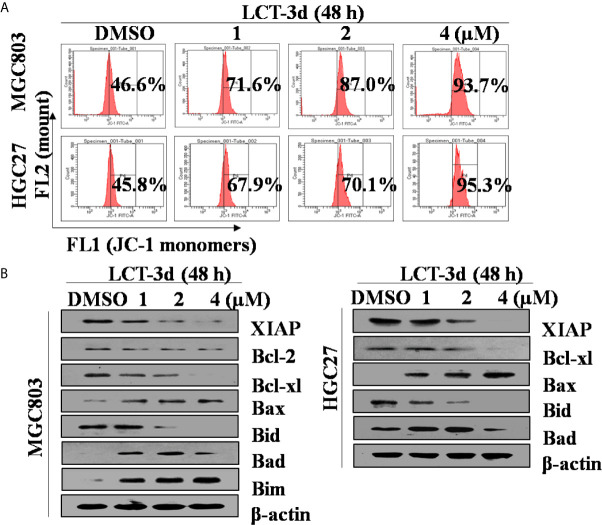
LCT-3d-induced apoptosis was associated with mitochondrial pathway in gastric cancer cells. **(A)** MGC803 cells and HGC27 cells were treated with various concentrations of LCT-3d for 48 h and the membrane potential was measured by JC-1 dye retention using flow cytometry. **(B)** Cells were treated as in **(A)** and the expression of Bax, Bad, Bim, Bid, Bcl-xL, Bcl-2, and XIAP proteins was determined by Western blotting.

To further explore the underlying mechanism of LCT-3d-induced apoptosis, the change of mitochondrial membrane potential (ΔΨ) and the level of proteins involved in apoptosis were investigated. It was found that LCT-3d reduced the mitochondrial membrane potential in MGC803 and HGC27 cells (**Figure 3A**). Furthermore, LCT-3d down-regulated the expression of anti-apoptotic proteins Bcl-2, Bcl-xl, Bid, and XIAP, and up-regulated the pro-apoptotic proteins Bax, Bad, and Bim in MGC803 cells (**Figure 3B**). These results demonstrated that LCT-3d induced apoptosis of gastric cancer cells via the mitochondrial pathway.

### DR5 Was Involved in LCT-3d-Induced Apoptosis in MGC803 Cells

**Figure 4:**
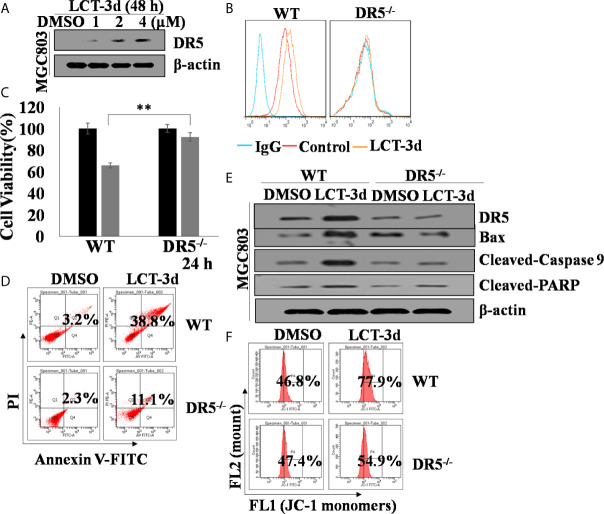
LCT-3d-induced apoptosis is associated with extrinsic pathway in gastric cancer cells. Both wild type and DR5^−/−^ MGC803 cells were treated with 4 μM LCT-3d or indicated concentration. **(A)** Western blotting assay showed reduced expression of DR5 in a dose-dependent manner in MGC803 cells. **(B)** MGC803 cells and DR5^−/−^ MGC803 cells were treated with 4 μM LCT-3d for 24 h and DR5 protein expressions in MGC803 cells determined by flow cytometry. **(C)** MTT assay showed the cell viability after treatment with 4 μM LCT-3d for 24 h. **P < 0.01, significantly different compared with control. **(D)** Flow cytometric analysis showed the ratios of apoptotic cells after treatment with 4 μM LCT-3d for 24 h. **(E)** Western blotting assay showed the expression of the apoptosis-related proteins after treatment with 4 μM LCT-3d for 24 h. **(F)** Flow cytometric analysis demonstrated the reduction of MMP (ΔΨ) after treatment with 4 μM LCT-3d for 24 h.

Then, the role of DR5 was evaluated on LCT-3d-induced apoptosis. It was found that LCT-3d significantly up-regulated the expression of DR5 in MGC803 cells (**Figure 4A**). To further confirm whether DR5 is necessary for LCT-3d-induced apoptosis, the DR5 knocked down MGC803 cells were used for investigation. Flow cytometry analysis showed that the expression of DR5 was reduced in DR5 knocked down MGC803 cells (**Figure 4B**). The LCT-3d-induced cell death was partially reversed in DR5 knocked down MGC803 cells (**Figures 4C, D**). The Cleaved-Caspase 9 and Cleaved-PARP were decreased in DR5 knocked down MGC803 cells compared with wild type MGC803 cells (**Figure 4E**). LCT-3d-induced mitochondrial depolarization was partially reversed in DR5 knocked down MGC803 cells (**Figure 4F**). Collectively, these results suggested that LCT-3d-induced apoptosis was mediated by DR5.

### LCT-3d Induced ROS Production and Activated Nrf2 Pathway in MGC803 Cells

**Figure 5:**
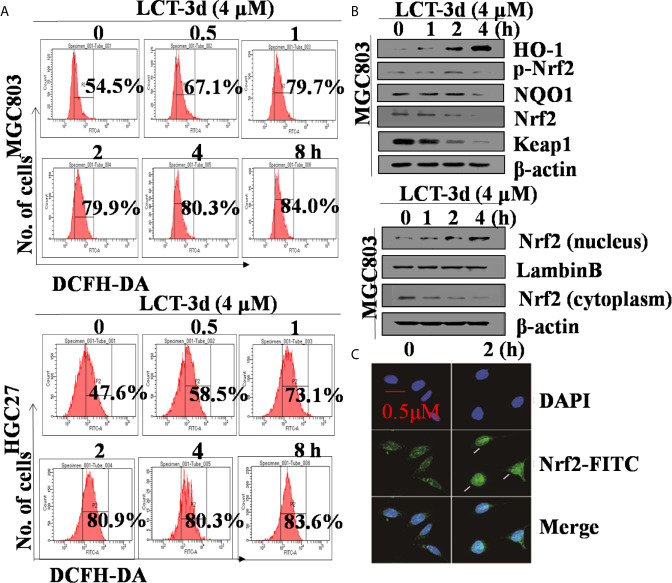
LCT-3d increased the level of ROS in gastric cancer cells. **(A)** MGC803 cells and HGC27 cells were treated with LCT-3d (4 μM) for indicated times and the level of ROS was detected by DCFH-DA with flow cytometry. **(B)** Western blotting assay showed Nrf2 nuclear-translocation and the changes of LCT-3d-induced protein expression at indicated time points. **(C)** MGC803 cells were treated with LCT-3d (4 μM) for 2 h. The treated and untreated samples are stained with Nrf2 antibody (Green) and DAPI (Blue) (magnification, 400×). The arrows indicate Nrf2 nuclear translocation.

To explore whether ROS was related to LCT-3d-induced apoptosis, the levels of ROS were detected after treatment with LCT-3d by flow cytometry. The results showed that the level of ROS was markedly increased in a time-dependent manner in MGC803 and HGC27 cells (**Figure 5A**). Next, we detected the role of Nrf2 in LCT-3d-induced ROS generation in MGC803 cells. The results showed that LCT-3d up-regulated p-Nrf2 and its downstream targets HO-1 and NQO1 in MGC803 cells (**Figure 5B**). Moreover, levels of Nrf2 in the nuclear fraction were significantly increased, whereas levels of Nrf2 in the cytoplasm were decreased, indicating that Nrf2 translocated into the nucleus and activated its downstream target genes (**Figure 5B**). To support this point, the immunofluorescence assay was conducted and similar result was observed (**Figure 5C**). These results suggested that LCT-3d induced the production of ROS and triggered the Nrf2 pathway to inhibit apoptosis of gastric cancer cells.

### LCT-3d-Induced Apoptosis Depends on ROS but Not Nrf2 in Gastric Cancer Cells

**Figure 6:**
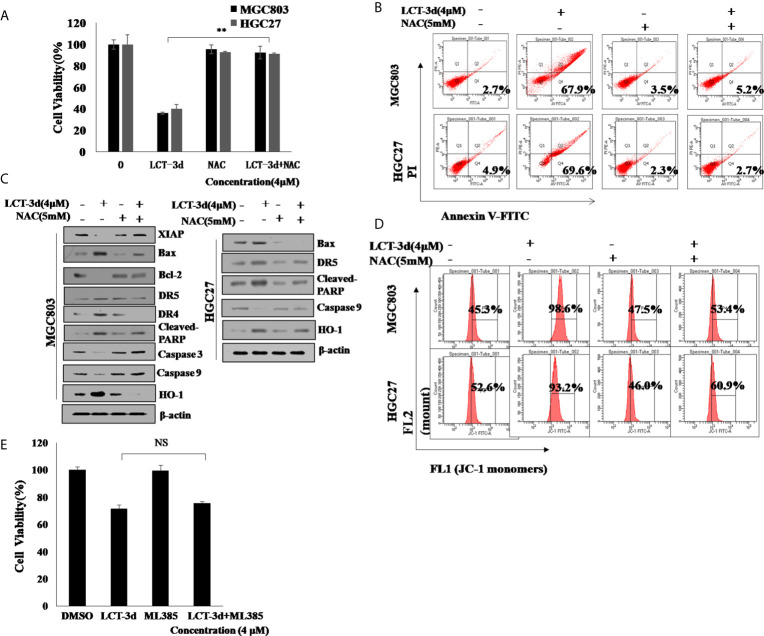
LCT-3d-induced apoptosis is associated with the rising ROS in gastric cancer cells. **(A)** MGC803 cells and HGC27 cells were pretreated with NAC (5 mM) for 1 h, followed by incubation with LCT-3d (4 μM) for 48 h. The effect of NAC on LCT-3d-induced cell death was analyzed by MTT assay. **P < 0.01, significantly different compared with control. **(B)** Flow cytometric analysis demonstrated the effects of NAC (5 mM) on LCT-3d (4 μM)-induced cell apoptosis of the gastric cancer cells. **(C)** Western blotting assay showed the effect of NAC (5 mM) on LCT-3d (4 μM)-induced change of protein expression at 48 h. **(D)** Flow cytometric analysis showed the effect of NAC (5 mM) on LCT-3d (4 μM)-induced loss of MMP (ΔΨ) in gastric cancer cells. **(E)** MGC803 cells pretreated with ML385 for 1 h followed by the treatment with LCT-3d (4 μM) for an additional 48 h. The pretreatment with ML385 rarely reversed the cell death in MGC803 and HGC27 cells.

To explore whether ROS contributed to the apoptosis induction, MGC803 and HGC27 cells were pretreated with NAC for 1 h followed by the treatment with LCT-3d (4 μM) for an additional 48 h. The pretreatment with NAC reversed the cell death in MGC803 and HGC27 cells (**Figures 6A, B**). The effect of ROS on cell apoptosis-related proteins was investigated by Western blotting. The results showed that NAC markedly reversed the changes of expression of apoptosis-related proteins, as well as abrogated mitochondrial depolarization. These results were similar in HGC27 cells to MGC803 cells (**Figures 6C, D**). However, the pretreatment with ML385 rarely reversed the cell death in MGC803 cells (**Figure 6E**). These results suggested that LCT-3d-induced apoptosis depended on ROS but not Nrf2 in gastric cancer cells.

## Discussion

The death rate of patients suffering from gastric cancer is increasing. The emergence of side effects and multi-drug resistance to chemotherapeutic drugs usually lead to the failure of chemotherapies (18). In this study, a new Indole compound LCT-3d was discovered, which had selective cytotoxicity against gastric cancer cells with no apparent cytotoxicity against non-malignant gastric epithelial cells. ROS level was detected by flow cytometry in gastric cancer cells. The cells pretreated with NAC for 1 h blocked the production of ROS and prevented the up-regulation of DR5, as well as mitochondria JNK-CHOP pathway (18). ROS triggered apoptosis by activating death receptors and the mitochondria pathway (19, 20). Mechanistically, the data demonstrated that LCT-3d induced gastric cell apoptosis through DR5-mediated mitochondrial pathway.

The activation of Caspase family proteins is an important event leading to cell apoptosis (21). At the early stage of apoptosis, Caspase 3 and Caspase 9 were activated, and they served as biomarker proteins of apoptosis (22). Caspase 3, the key execution enzyme, was activated by Caspase 9, which is the primary initiator Caspase (23). The cleavage of PARP was regarded as an important indicator of apoptosis (24). Therefore, the expression of these proteins was measured by Western blotting. The results showed the activation of Cleaved-Caspase 3/9 and Cleaved-PARP after treatment with LCT-3d. The reversion of the process by Z-VAD-FMK confirmed that LCT-3d induced apoptosis dependent on Caspases.

It has long been reported that mitochondria is an important factor in the process of apoptosis (25). To further confirm whether LCT-3d treatment activated mitochondrial pathway, we detected mitochondria-related proteins in gastric cancer cells. Bcl-2 family proteins are the major regulators and effectors of the mitochondrial apoptotic pathway, which include anti-apoptotic proteins such as Bcl-2 and pro-apoptotic proteins such as Bax (26–29). The oligomerization of Bax and Bak proteins promote the increase of the mitochondrial membrane permeability, which leading to the release of cytochrome c into the cytosol, subsequently cell apoptosis occurred through Caspase cascade. In this work, mitochondrial membrane potential and the levels of Bcl-2 and Bcl-xL were decreased upon LCT-3d treatment. Meanwhile, pro-apoptotic protein Bax, Bad, and Bim were increased after LCT-3d treatment. Collectively, the findings demonstrated that LCT-3d induced apoptosis of gastric cancer cells *via* mitochondrial pathway.

The role of DR5 in LCT-3d-induced apoptosis was investigated. MTT assay showed that LCT-3d-induced cell death was partially reversed in MGC803-DR5^−/−^ cells, suggesting that DR5 is an important factor for inducing cell apoptosis upon LCT-3d treatment. Moreover, compared with wild type MGC803 cells, the apoptotic rate and the decline of MMP as well as the cleavage of PARP and Caspase 9 were reversed in MGC803-DR5^−/−^ cells. These results demonstrated that activation of mitochondrial pathway was associated with DR5.

ROS can trigger the mitochondria-mediated intrinsic apoptotic pathway (19, 30). In this work, the elevating ROS in the process of LCT-3d-induced apoptosis was observed. In addition, NAC markedly inhibited the activation of Caspase 3/9, the up-regulation of DR5, the modulation of Bcl-2 family proteins, as well as the cancellation of MMP decrease. These results suggested that NAC blocked the effect of ROS on gastric cancer cells, indicating that ROS triggered cell death through DR5-mediated mitochondrial apoptosis pathway (31).

ROS are the key molecules in the process of apoptosis (32, 33). It is reported that moderate level of ROS can increase the ability of cell survival (34). It is well known that Nrf2 can protect cells from injury by oxidative stress through separating from Keap1 and subsequently translocating into the cell nucleus (35, 36). In this study, it was confirmed that the elevating ROS activated Nrf2, which then translocated into the nucleus, subsequently up-regulated its downstream target genes such as HO-1, NQO1, leading to inhibition of apoptosis, which is in consistent with the previous work (31, 37).

## Conclusion

**Figure 7:**
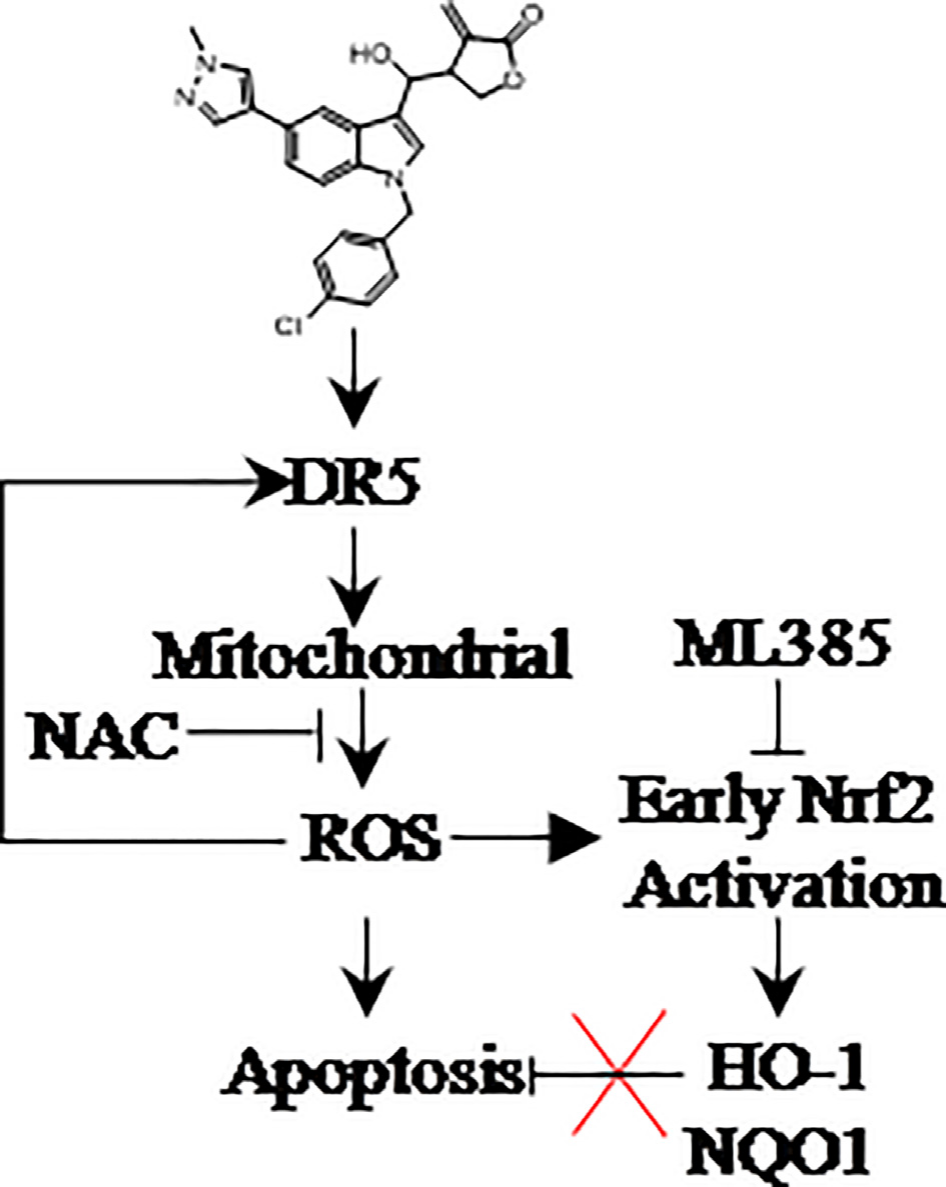
LCT-3d selectively kills gastric cancer cells but not normal cells *via* inducing ROS triggered apoptosis by activating death receptors and the mitochondria pathway. ROS scavenger NAC can block cell death, while Nrf2 inhibitor ML385 has no effect on LCT-3d-induced cell death. The promotion of ROS-induced apoptosis by LCT-3d depends on the up-regulation of DR5 but not the inactivation of Nrf2.

A novel Indole derivative LCT-3d was found to induce apoptosis in gastric cancer cells through DR5-mediated mitochondrial apoptotic pathway (13, 31). LCT-3d modulated the Bcl-2 family proteins and Cleaved-Caspases 3/9, and resulted in cell apoptosis in MGC803 and HGC27 cells. Nrf2 played an important role in protecting gastric cancer cells from the injury of oxidative stress after LCT-3d treatment, but it could not reverse LCT-3d-induced cell apoptosis (**Figure 7**). This study suggested that LCT-3d could be a potential lead compound for the development of anti-gastric cancer agents.

## Data Availability Statement

The original contributions presented in the study are included in the article/supplementary material. Further inquiries can be directed to the corresponding authors.

## Author Contributions

MW, XW, LY, and Z-YH performed majority experiments, analyzed data, and wrote original draft. XL, XM, and C-TL synthesized the compound LCT-3d. G-YK and YC knocked down the DR5. C-YJ, H-WX, and ZW conceived and designed the experiments and revised the draft of manuscript. All authors contributed to the article and approved the submitted version.

## Funding

The work was supported by the National Natural Science Foundation of China (Project No. 81973529 for C-YJ), “13th five-year plan of China”: Major Projects of National Science and Technology on New Drug Creation and Development (Project No. HX2018ZX09711001-005-026 for HX), and Fang’s family (Hong Kong) foundation (ZW).

## Conflict of Interest

The authors declare that the research was conducted in the absence of any commercial or financial relationships that could be construed as a potential conflict of interest.

The reviewer JH declared a shared affiliation with one of the authors, G-YK, to the handling editor at time of review.
