# Corrigendum: LCT-3d induces oxidative stress-mediated apoptosis by upregulating death receptor 5 in gastric cancer cells

**DOI:** 10.3389/fonc.2025.1590809

**Published:** 2025-05-29

**Authors:** Menglin Wang, Xinxin Wu, Lu Yu, Zi-yun Hu, Xiaobo Li, Xia Meng, Chun-tao Lv, Gi-Young Kim, Yung Hyun Choi, Zhenya Wang, Hai-Wei Xu, Cheng-Yun Jin

**Affiliations:** ^1^ Key Laboratory of Advanced Technology for Drug Preparation, Ministry of Education, School of Pharmaceutical Sciences, Zhengzhou University, Zhengzhou, China; ^2^ Department of Marine Life Sciences, Jeju National University, Jeju, Republic of Korea; ^3^ Department of Biochemistry, College of Oriental Medicine, Dong-Eui University, Busan, Republic of Korea; ^4^ State Key Laboratory of Esophageal Cancer Prevention & Treatment, Zhengzhou University, Zhengzhou, China

**Keywords:** LCT-3d, DR5, reactive oxygen species, Nrf2, apoptosis, gastric cancer

In the published article, an author name was incorrectly written as “Zhengya Wang”. The correct spelling is “Zhenya Wang”.

In the published article, there was an error in **Figure 2** as published. During the post-submission stage, while organizing and editing the figures, we failed to conduct a thorough check, resulting in a fundamental error in **Figure 2** of the manuscript: the duplication of Western Blot bands (strip 2 of MGC803 cells and strip 4 of HGC-27 cells in **Figure 2**. The corrected **Figure 2** and its caption LCT-3d triggered Caspase mediated apoptotic pathway in gastric cancer cells appear below.

**Figure 2:**
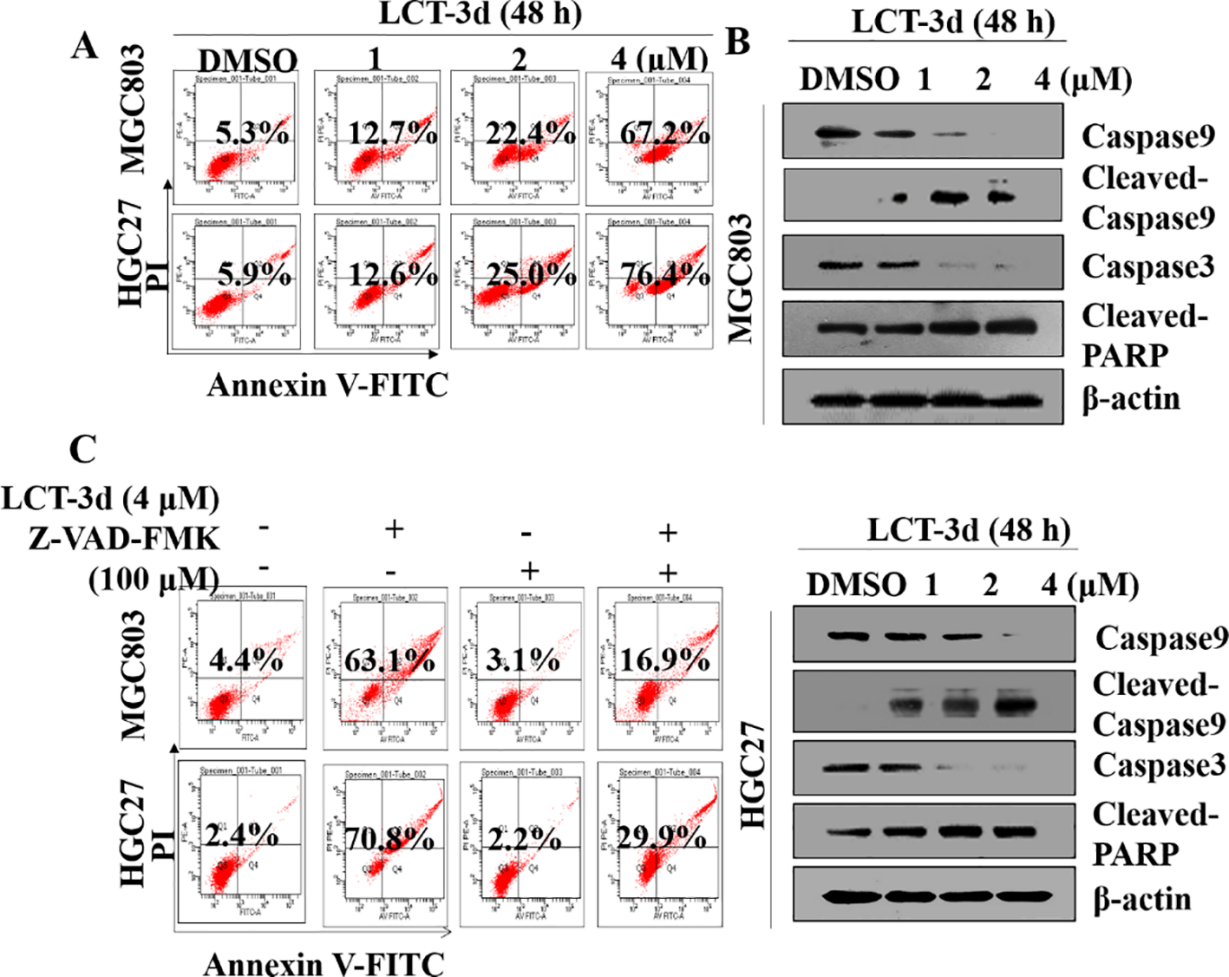
LCT-3d triggered Caspase mediated apoptotic pathway in gastric cancer cells. **(A)** MGC803 cells and HGC27 were cells treated with various concentrations of LCT-3d for 48 h and apoptosis analyzed by flow cytometry. **(B)** Cells were treated as in **(A)** and the expression of Cleaved-Caspase and Cleaved PARP was analyzed by Western blotting. **(C)** MGC803 cells and HGC27 cells were pretreated with a pan-Caspase inhibitor, Z-VAD-FMK (100 µM) for 1 h, followed by incubation with LCT-3d (4 µM) for 48 h. Flow cytometric analysis on the effect of Z-VAD-FMK on LCT-3d-induced cells apoptosis.

The authors apologize for these errors and state that this does not change the scientific conclusions of the article in any way. The original article has been updated.

